# Death from a Medico-Legal Aspect.—III

**Published:** 1911-12-02

**Authors:** 


### DEATH FROM A MEDICO-LEGAL ASPECT.—III.

(*Continued from p.* 176.)

### (i) Saponification of the Body.

It often becomes a subject of much importance to ascertain how long the body has lain in the water. The saponification of the body, or the formation of adipocere, occasionally occurs at a long period after death. This change is wholly chemical, and consists in the union of the fatty acids with ammonia. It has most frequently been observed in bodies that have lain a long time in water, or in very damp soil where moisture has continuously acted upon a putrefying corpse. There are some other conditions under which adipocere is formed, but the two mentioned are the only ones definitely known. Its formation to any considerable extent usually requires a long time, though it may begin to form at a rather early period. Adipocere is a fatty substance, generally of a yellow colour, occasionally of a pure white. It is unctuous or soapy, brittle, and soft to cut. It melts at different degrees of temperature, some requiring no more than 200° F. As it is due to the checking of the colliquative putrefaction, it occurs more readily in the bodies of fat people than in the lean, and children present the change more readily than do adults. Water, in any situation, will produce this state, although running water has been found to do so more rapidly. A question how long a time is necessary to cause this change to take place was made the subject of a very interesting legal inquiry.

### A Curious Case of 1805.

At the Lent Assizes held at Warwick in the year 1805 the following cause came before the court. A gentleman named Meecham, who was insolvent, left his own house with the intention (as was presumed from his recent conduct and conversation) of destroying himself. Five weeks and four days after that period his body was found floating down a river three miles from Birmingham, the place where he resided. The face was disfigured by putrefaction, and the hair separated from the scalp by the slightest pull; but the other parts of the body were firm and white without any putrefactive appearance.

The clothes were unaltered, but the linen was exceedingly rotten. On examining the body, it was found that the lower part of the abdomen and the glutei muscles were converted into adipocere. "A commission of bankruptcy having been taken out against the deceased a few days after he left home, it became an important question in the interest of his family to ascertain whether or not he was living at that period. From the changes which the body had sustained it was presumed that he had drowned himself on the day he left home, and to corroborate this presumption the evidence of Dr. Gibbes of Bath was requested, who from his experiments on this subject was better acquainted with it than any other. He stated, on the trial, that he had procured a small quantity of this fatty substance by immersing the muscular parts of animals in water for a month, and that it requires five or six weeks to make it in any large quantity. Upon this evidence the jury were of opinion that the deceased was not alive at the time the commission was taken out, and the bankruptcy was accordingly superseded." (Male, p. 192.)

Adipocere is a very permanent body, and may last for twenty years and upwards. The time required for the formation of adipocere is naturally variable, depending, as it does, on so many internal and external conditions. It has been held that two or three months' submersion in water is about the shortest time in which it is formed; traces, however, have been found in from four to five weeks. In moist earth it takes longer to form—eight to twelve months. It may be accepted that in temperate climes indications of saponification in bodies placed under circumstances favourable to its production may be met with one month after death; for the whole of the soft parts to be converted into adipocere many years are required.

### An Indian Example.

In hot climates the process may be marvellously rapid. The following cases reported by Mackenzie (The *Indian Med. Gaz.*, 1889) occurred in Calcutta: (1) A male Hindoo was killed by the kick of a horse, and was buried the following day. Four days after burial the body was exhumed in order that an inquest might be held; it was in an advanced state of saponification externally, the heart and liver being also saponified. (2) A young European was drowned in the river Hooghly, his body being recovered seven days afterwards. It was in an advanced state of saponification externally; the lungs, heart, liver, kidneys, stomach, and intestines were also saponified, and what is very curious is that the stomach contained undigested food—flesh and potatoes—of which the flesh was entirely saponified, the potatoes not being altered in the least. Other instances of early conversion into adipocere are recorded as occurring in India; in one case the body was saponified externally and internally in two days.

### Children and Women.

Other things being equal, the bodies of children putrefy more rapidly than those of adults and aged persons, and the bodies of old persons more rapidly than those of ordinary adults. According to Orfila, putrefaction is more rapid in women than in men. He attributes this to the greater quantity of fat they contain—an explanation which, though not quite satisfactory, agrees with the fact that the corpulent putrefy more readily than the lean and emaciated. Casper, who disputes the influence of sex, observes that the bodies of women dying during or soon after childbirth putrefy very rapidly. Putrefaction takes place more speedily in bodies filled with fluid. In low fevers, the extremities are attacked before the trunk has ceased to live.

